# Estimation of Blood Pressure in the Radial Artery Using Strain-Based Pulse Wave and Photoplethysmography Sensors

**DOI:** 10.3390/mi9110556

**Published:** 2018-10-29

**Authors:** Yu-Jen Wang, Chia-Hsien Chen, Chung-Yang Sue, Wen-Hsien Lu, Yee-Hsuan Chiou

**Affiliations:** 1 Department of Mechanical and Electromechanical Engineering, National Sun Yat-Sen University, Kaohsiung 80424, Taiwan; a22325498@gmail.com; 2 Smart Microsystems Technology Center, Industrial Technology Research Institute, Tainan 70955, Taiwan; cysue@itri.org.tw; 3 Department of Pediatrics, Kaohsiung Veterans General Hospital, Kaohsiung 81362, Taiwan; lu6802@gmail.com (W.-H.L.); yhchiou@vghks.gov.tw (Y.-H.C.); 4 Faculty of Medicine, National Yang-Ming University, Taipei 11221, Taiwan

**Keywords:** continuous blood pressure measurement, radial artery, strain gauge, photoplethysmography, pulse transit time

## Abstract

Blood pressure (BP) is a crucial indicator of cardiac health and vascular status. This study explores the relationship between radial artery BP and wrist skin strain. A BP estimation method based on the physical model of wrist skin tissues and pulse wave velocity (PWV) is proposed. A photoplethysmography (PPG) sensor and strain gauge are used in this method. The developed strain-based pulse wave sensor consists of a pressing force sensor, which ensures consistent pressing force, and a strain gauge, which measures the cardiac pulsation on the wrist skin. These features enable long-term BP monitoring without incurring the limb compression caused by a cuff. Thus, this method is useful for individuals requiring continuous BP monitoring. In this study, the BP of each participant was measured in three modes (before, during, and after exercise), and the data were compared using a clinically validated sphygmomanometer. The percentage errors of diastolic and systolic BP readings were, respectively, 4.74% and 4.49% before exercise, 6.38% and 6.10% during exercise, and 5.98% and 4.81% after a rest. The errors were compared with a clinically validated sphygmomanometer.

## 1. Introduction

Blood pressure (BP), a vital sign used to monitor the functioning of human body, is a crucial indicator of cardiac health and vascular status. BP readings and pulse wave patterns reflect specific physiological responses; continuous readings of these two vital signs may be used to examine an individual’s physiological condition and identify signs of chronic diseases. The variability of BP can be influenced by various external factors (including a person’s emotions, exercise, and dietary habits). Accordingly, BP data from a single measurement may vary and cause misjudgment of a patient’s health status. Continuous monitoring of BP during each cycle of pulsation facilitates detection of abnormal BP changes and generates data that immediately and accurately indicate a person’s physiological condition. These data can be collected using two methods: The direct measurement method (the invasive method) and the indirect measurement method (the noninvasive method). The invasive method must be conducted by professional nurses, and involves insertion of an arterial line directly into an artery. An advantage of this method is that accurate BP readings can be obtained immediately. However, it can be employed only during a surgery or in an intensive care unit.

The first noninvasive BP measurement [1] was developed by Von Bausch and Riva-Rocci, and features a water-filled bag and a cuff that together serve as a sensing device. This BP measurement system is still applied in contemporary practice. Noninvasive BP measurements are typically read using the auscultatory method or the oscillometric method. The noninvasive measurement method tends to cause cuff-inflation-induced discomfort for the patient in the measured area, and the method cannot be used for consecutive measurements. Blood pulse wave–sensing devices that are suitable for noninvasive continuous monitoring can be classified as optical [2,3] or mechanical [4,5,6,7,8,9,10,11]. Optical monitoring is known as photoplethysmography (PPG), wherein light is projected onto the user’s skin to detect cyclic changes in blood volume per unit area of the artery. The cyclic changes are caused by cardiac pulsation, and the light intensity varies according to changes in the blood volume. In mechanical monitoring, a strain sensor is attached to a skin surface over an artery to sense changes in arterial pulse pressure caused by cardiac pulsation [12].

Noninvasive long-term pulse and BP monitoring can be achieved using the mechanical [13,14,15], optical [16,17,18], and pulse transit [19,20] methods. In its preliminary design, the mechanical method for continuous BP monitoring involved placing a tension sensor on the skin surface over a carotid or radial artery. The deformation of the sensor was then observed through skin-strain changes caused by cardiac pulsation to measure arterial pulse waves and BP. The tension sensor approach is the predecessor of today’s tonometry. The mechanical method can also be performed using a magnetoelastic and bendable bilayer sensor where the bilayer is enveloped by a coil [15]. For this BP waveform measurement method, the sensor is attached to the surface of the patient’s neck, over the carotid artery, and changes in the magnetic permeability of the coil are monitored.

The optical method is generally based on light transmission [16] or reflection [17]. In contrast to the reflection-based approach, the transmission-based approach can only be applied to certain body areas, such as fingers and earlobes. This is because the light projected for transmission cannot penetrate thick tissues sufficiently. The reflection-based approach is more widely adopted, and its application is less limited. In this study, we proposed applying a strain gauge and an optically reflective PPG to estimate BP continuously and without using a cuff. The PPG is placed on a user’s fingertip to measure the reflected light. From variations in the reflected light, pulsatile variation in the blood volume during cardiac pulsation can be observed. During systole, when the pulse wave arrives, blood volume increases in the tissues, and because of absorption, the received light intensity decreases [18]. Measurements of light intensity variations can then be used to generate a waveform graph of cyclic trends.

Another method for continuous monitoring of BP is based on pulse transit time [19,20]. This approach involves placing two sensors on two body areas to measure the pulse wave velocity (PWV) or the pulse transit time (PTT). Subsequently, the relationship of the measured PWV or PTT with BP is derived. The temporal resolution and effects of measurement noise for PWV calculation have been discussed [21]. The present study employed a novel hybrid of mechanical and optical sensors for continuous monitoring of BP and PWV. A physical model for calculating BP based on pulse wave measurement was incorporated into the research procedures in which BP equations were derived based on PTT and skin strain to achieve accurate and continuous BP monitoring.

## 2. BP Estimation Method and Model

**Figure 1:**
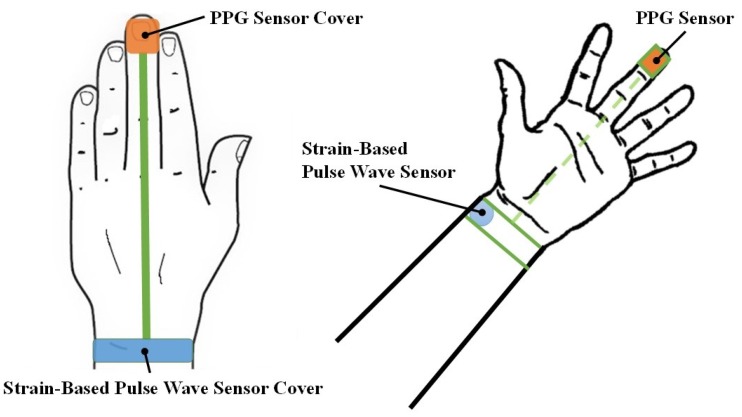
Schematic the proposed continuous blood pressure (BP) monitoring system.

This study devised a continuous and cuffless BP monitoring system (Figure 1). Cardiac pulsation causes changes in blood volume in the radial artery, and these changes lead to arterial dilation and contraction, thus inducing strain on the surface tissue. The proposed system consists of a strain-based pulse wave sensor that measures the aforementioned strain changes, facilitating determination of the effects of changes in BP on the skin surface. To monitor BP and PTT, the present study established a physical model in which the skin strain induced by the radial artery is measured. A strain-based pulse wave sensor was integrated with a PPG sensor to measure PTT and skin strain. These measurements were then used to formulate equations for BP estimation.

### 2.1. Establishment of Models for Estimating BP

Human skin and blood vessel tissues are thick vessels [22]; therefore, this study applied the thick vessel theory to evaluate stress and strain on blood vessel walls resulting from the external and internal pressure. PWV-related theories were also applied, and equations for estimating BP were formulated accordingly. The influence of intravascular pressure ($p_{i}$) on the external vessel wall can be expressed as:

(1) $$p_{i}=\frac{1}{2{r_{i}}^{2}}\cdot \left[\frac{-E_{eff}\cdot \nu }{\left(1-2\nu \right)\left(1+\nu \right)}\cdot \varepsilon_{\theta \theta }\cdot \left({r_{o}}^{2}-{r_{i}}^{2}\right)+p_{o}\left({r_{o}}^{2}+{r_{i}}^{2}\right)\right],$$

where $r_{i}$ denotes the inner diameter of the blood vessel, $r_{o}$ the vascular diameter plus the distance between the vessel and the skin surface, $p_{o}$ the pressing force applied to the skin surface; $\varepsilon_{\theta \theta }$ the circumferential strain, $E_{eff}$ the equivalent elasticity coefficient between the blood vessel and the skin, and ν is the Poisson’s ratio. The thick vessel theory is used to modify $r_{i}$ to ri+h2 in the Moens–Korteweg equation, and the following equation can thus be obtained:

(2) Vpw2=Eeff·h2·ρ·(ri+h2),

where $V_{pw}$ denotes the PWV, *h* the distance between the blood vessel and skin surface, and ρ the blood density.

Substituting $V_{pw}$ for $E_{eff}$, in Equation (1), the strain on the skin surface induced by diastolic BP (DBP; $p_{iD}$) can be calculated as a function of $\varepsilon_{\theta \theta D}$. *D* and *S* denote diastolic and systolic BP, respectively. Consequently, Equation (1) can be rewritten as:

(3) piD=1ri2·[−Vpw2·ρ·(ri+h2)·νh·(1−2ν)(1+ν)·εθθD·(ro2−ri2)+po(ro2+ri2)].

The strain induced by DBP ($\varepsilon_{\theta \theta D}$) appears to arise from an initial strain ($\varepsilon_{\theta \theta r}$) caused by a constant intravascular pressure ($p_{ir}$). Moreover, the PWV ($V_{pw}$) can be expressed as $\frac{L}{PTT}$, where L denotes the distance between the finger and wrist sensors (i.e., the distance between the finger and radial artery). Therefore, Equation (3) can be expressed as:

(4) piD=1ri2·[−(LPTT)2·ρ·(ri+h2)·νh·(1−2ν)(1+ν)·(εθθD−εθθr)·(ro2−ri2)]+pir

To measure systolic BP (SBP), which appears to reflect DBP with additional external BP, the following equation is developed:

(5) piS=1ri2·[−(LPTT)2·ρ·(ri+h2)·νh·(1−2ν)(1+ν)·(εθθS−εθθD)·(ro2−ri2)]+piD

where $p_{iS}$ denotes SBP and $\varepsilon_{\theta \theta S}$ the strain caused by the SBP on the skin.

Subsequently, Equations (4) and (5) can be rewritten as Equations (6) and (7), respectively.

(6) $$p_{iD}=C_{1}\cdot {\left(\frac{1}{PTT}\right)}^{2}+C_{2}$$

where C1=1ri2·−L2·ρ·(ri+h2)·νh·(1−2ν)(1+ν)·(εθθD−εθθr)·(ro2−ri2); C2=pir

(7) $$p_{iS}=C_{3}\cdot \frac{\left(\varepsilon_{\theta \theta S}-\varepsilon_{\theta \theta D}\right)}{PTT^{2}}+p_{iD}+C_{4}$$

where C3=1ri2·−L2·ρ·(ri+h2)·νh·(1−2ν)(1+ν)·(ro2−ri2) and $C_{4}$ is an offset constant.

$C_{1}$, $C_{2}$, $C_{3},$ and $C_{4}$ are calibration parameters for an individual’s data (e.g., vascular diameter and elasticity coefficients of tissues). In the experiments, calibration was performed by incorporating a strain-based pulse wave sensor and a PPG sensor and using before and after exercise BPs from a clinically validated sphygmomanometer. During calibration, the values of PTT and strain difference $(\varepsilon_{\theta \theta S}-\varepsilon_{\theta \theta D}$≡$\varepsilon_{\theta \theta S-D}$) for the individual were determined, enabling calculation of the regression line and estimation of the DBP and SBP. The least-mean-square method was adopted to acquire calibration parameters.

### 2.2. Calculation of Pressing Force

**Figure 2:**
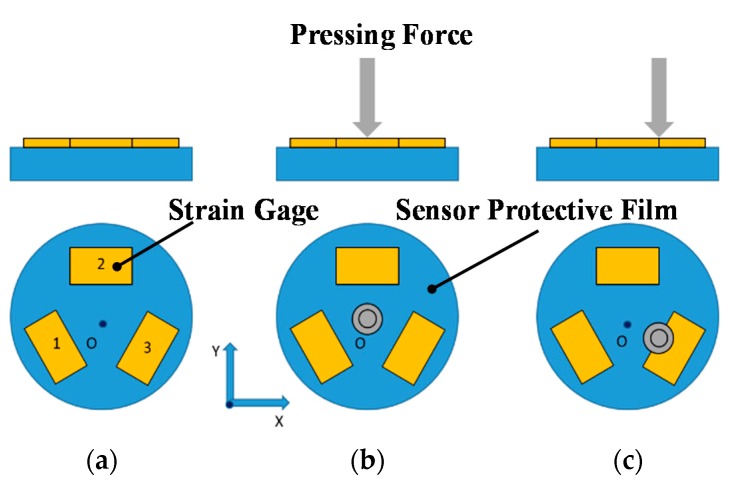
Schematic of the proposed pressing force sensor. (**a**) No force applied. (**b**) Centric force applied. (**c**) Eccentric force applied.

During BP measurement, pressing force and location variation may influence the applicability of the calibration parameters in the BP equation, thereby affecting the accuracy of BP estimation. In this study, a pressing force sensor was employed to calculate the pressing force applied to a certain point and ensure consistent measurement conditions. Specifically, the pressing force sensor, comprising an elastic body and three strain gauges arranged in an equilateral triangle, was employed to identify the magnitude and location of the pressing force. Figure 2 illustrates the proposed pressing force sensor for situations in which no, centric, and eccentric pressing forces are applied. Based on the strain detected by the three sensors, the magnitudes and locations of the resultant pressing forces can be calculated.

**Figure 3:**
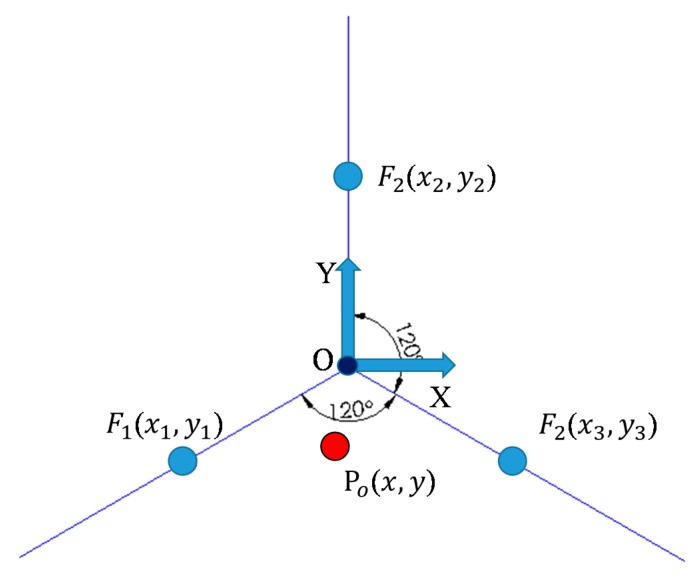
Schematic of the distribution of the resultant pressing force.

Based on the three strain gauges, pressing force (*F*_1~3_) components are acquired (Figure 3). According to forces (*F*_1~3_) and corresponding locations, the location of the resultant pressing force is evaluated using Equations (8) and (9).

(8) $$x=\frac{F_{1}x_{1}+F_{2}x_{2}+F_{3}x_{3}}{p_{o}}$$

(9) $$y=\frac{F_{1}y_{1}+F_{2}y_{2}+F_{3}y_{3}}{p_{o}}$$

where x and y denote the force coordinates, and $p_{o}$ is the sum of *F*_1~3_.

## 3. Device Design

**Figure 4:**
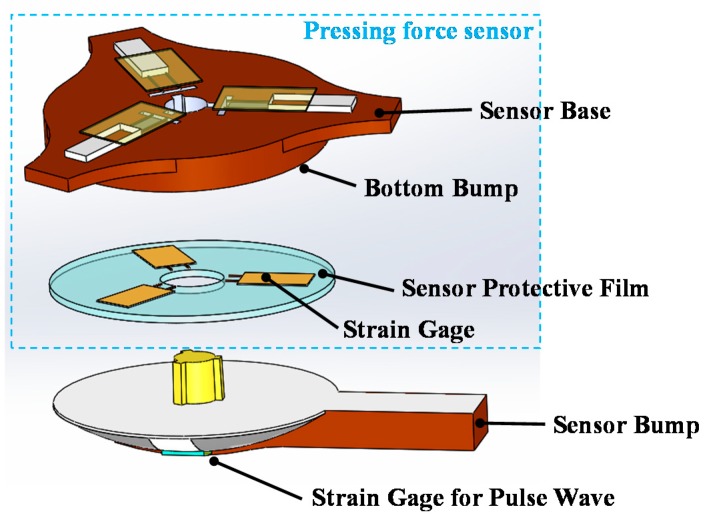
Schematic of the proposed strain-based pulse wave sensor.

**Figure 5:**
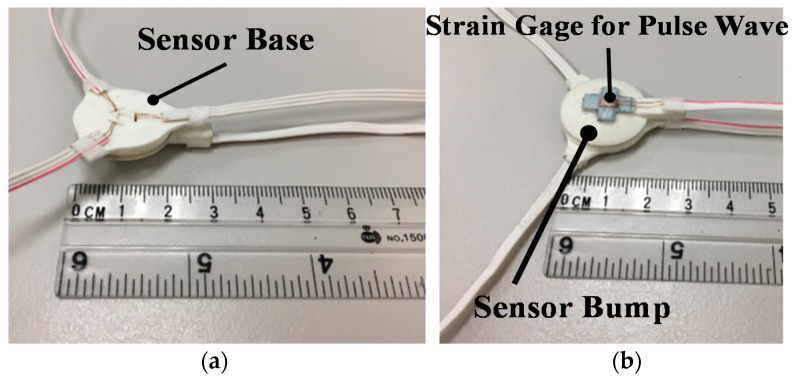
Photographs of the proposed strain-based pulse wave sensor. (**a**) Top of the sensor (through which the conducting wires are channeled). (**b**) Bottom of the sensor (where strain gauges are attached).

The strain-based pulse wave sensor is composed of a strain gauge, which measures radial artery pulse waves, and a pressing force sensor, which detects pressing force in the measurement (Figure 4). The pressing force sensor is used to ensure the repeatability of the pressing force in the measurement. The pressing force sensor comprises a sensor base, sensor protective film with three stain gauges, and sensor bump. The upper sensor base features three wire holes that the wires of the strain gauges located below can pass through and thereby be covered with polyimide films that protect subjects from direct contact with the wires. Three circular bumps at the bottom of the sensor base strengths the gauge sensitivity. A soft silicon film serves as a flexible buffer film to enlarge the pressing force signal. The sensor bump in the bottom features a guide shaft that is used to assemble the bump with the other components. In addition, a cross-shaped protrusion is designed on the sensor bump to facilitate positioning. Figure 5 displays photos of the proposed strain-base sensor. The strain gauge for pulse wave measurement is attached to the wrist skin with pressing force provided by the wrist belt.

## 4. Methods

**Figure 6:**
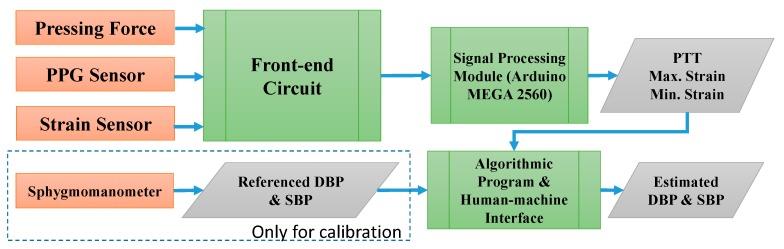
Signal flowchart for BP estimation.

**Figure 7:**
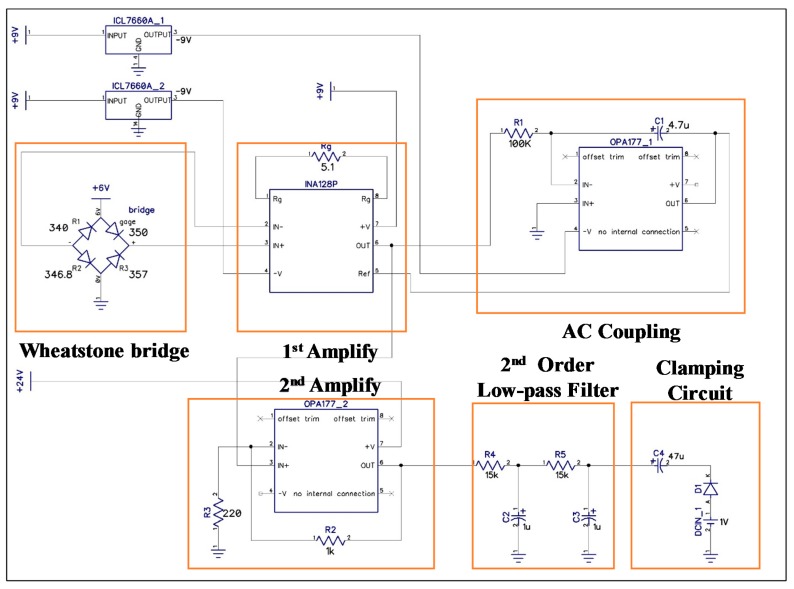
Schematic of the front-end circuit designed for the strain-based pulse wave sensor.

**Figure 8:**
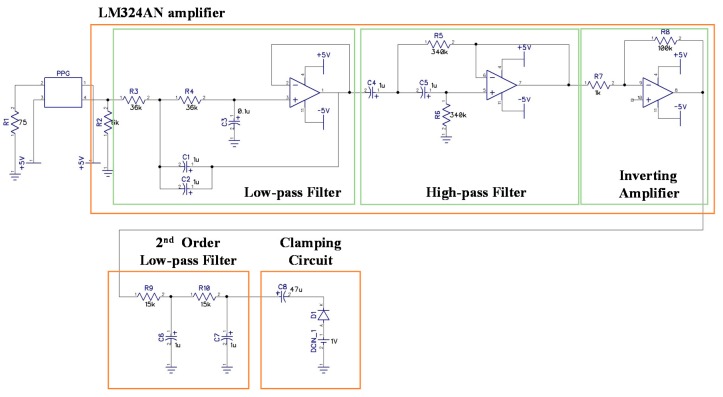
Schematic of the front-end circuit designed for the photoplethysmography (PPG) sensor.

PPG sensors were integrated with the proposed strain-based pulse wave sensor to obtain adequate PTT and skin-strain data for estimating BP. Thus, at this stage, the BP measuring system consists of four components: A front-end circuit, signal processing module, algorithmic program, and human–machine interface (listed according to the order of usage; Figure 6). The front-end circuit comprises a strain-sensing circuit and a PPG readout circuit, schematic diagrams of which are presented in Figure 7 and Figure 8, respectively. In the experiments, the signal processing module, realized by Arduino MEGA 2560 (Arduino, Ivrea, Italy) with the sample rate of 5 kHz, was used to receive signals from the front-end circuit. Estimations of the calibration parameters and BP were performed in an algorithmic program using LabVIEW (8.0, National Instruments, Austin, TX, USA). Skin strain was measured on the basis of output voltage signals from a Wheatstone bridge circuit. However, because the strain value produced by radial artery pulses was 10^−5^ to 10^−4^, the output voltage signals in the model were only a few microvolts. Therefore, an instrumentation amplifier (INA 128P, Texas Instruments, Dallas, TX, USA) was employed for first-stage signal amplification. Following first-stage signal amplification, the reference potential was modified through an alternating current coupling circuit to eliminate signal offset, and a second amplification circuit was used to obtain a higher signal amplification ratio. The average human heart rate is 60–100 beats per minute when an individual is in a state of calm, and the rate is higher when an individual is exercising. Thus, a second-order low-pass filter with a cutoff frequency of 10 Hz was used to filter out high-frequency noise, and a clamping circuit was employed to adjust the signal level. In the PPG readout circuit, an LM324AN amplifier (Texas Instruments, Dallas, TX, USA) was adopted to amplify PPG signals and thereby achieve band-pass filtering. A second-order low-pass filter with cutoff frequency of 10 Hz was also installed to filter out high-frequency noise, and a clamping circuit was used to adjust the signal level to comply with the range of the Arduino analog input port.

**Figure 9:**
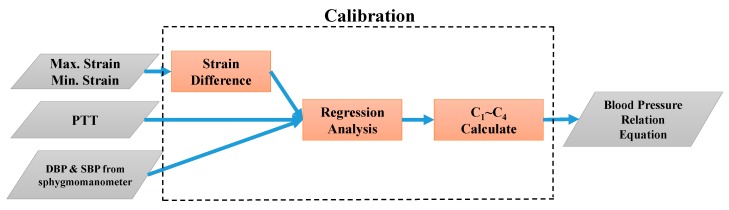
Flowchart of program calibration via LabVIEW.

**Figure 10:**
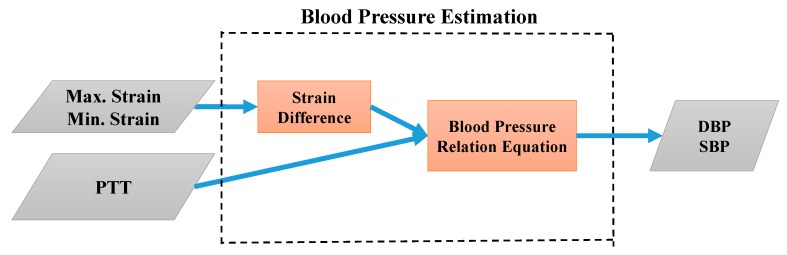
Flowchart of BP estimation via LabVIEW.

In this study, the analog strain and PPG signals were converted into digital signals using an Arduino microcontroller in the signal processing module. The calculated PTT and the maximum and minimum strain values were input into LabVIEW, a graphical programming platform, to facilitate the development of an algorithmic program and a human–machine interface. In the first stage, the calibration parameters were evaluated on LabVIEW as depicted in Figure 9. In the second stage, the BP equations were used to perform continuous BP estimation, the process of which is illustrated in Figure 10.

**Figure 11:**
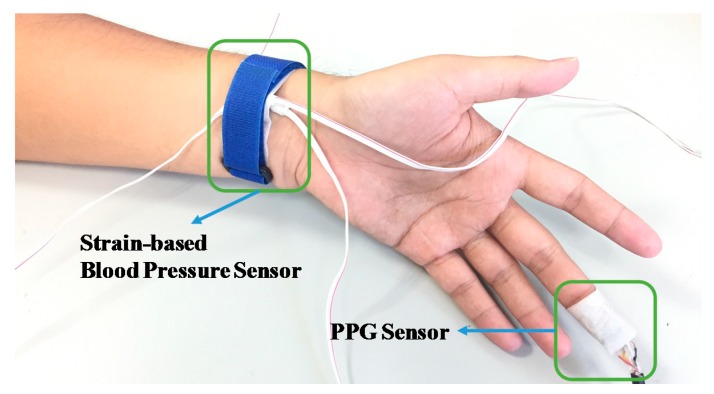
Photograph of a participant’s hand with the proposed high-sensitivity BP monitoring system.

**Figure 12:**
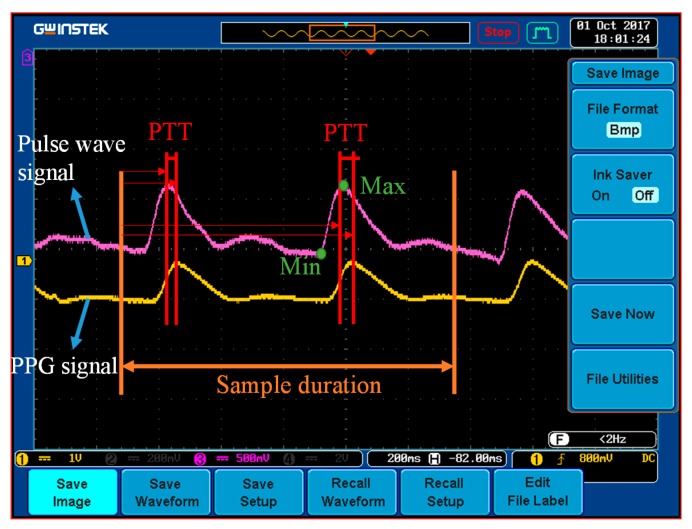
Photograph of an oscilloscope display featuring PPG and pulse wave signals.

This study was subjected to ethical review and approved by the Institutional Review Board of the Kaohsiung Veterans General Hospital of Taiwan. The participants were informed about the process and purpose of the study in advance. The measurement experiment of the present study was divided into two stages. The objective of the first stage was to calculate calibration parameters. Two sets of data, reflecting measurements before and during exercise, were used to estimate the calibration parameters based on Equations (6) and (7). Each participant was assisted in identifying the location of their radial artery, wrapping the proposed strain-based pulse wave sensor around their wrist, and affixing the PPG sensor on the pulp of their middle finger (Figure 11). After the sensors had been positioned, the participants’ PPG and wrist-strain signals were continuously monitored, and a clinically validated sphygmomanometer (WatchBP O3, Microlife, Microlife AG, Widnau, Switzerland) was simultaneously employed to measure BP values from the participant’s other hand. The reference BPs were measured during exercise (as well as before and after). The PTT was estimated based on the time difference between the two peaks of the PPG and pulse wave signals, as illustrated in Figure 12. In general, the distance between the wrist and the fingertip among the participants was approximately 20 cm, and the PWV was approximately 10 m/s; accordingly, the 5-kHz sample rate of the microprocessor (Arduino MEGA 2560) provided 1–2-mmHg resolution of the estimated BP. The peak times were acquired from the derivatives of the PPG and pulse wave and were calculated in the microprocessor.

After parameter calibration in the first stage, the participants were requested to rest for 5–10 min to allow their BPs to return to normal levels before the second stage of the experiment. In the second stage, the participants’ BP values were estimated using the calibration parameters and Equations (6) and (7). These estimations were performed as determined in the previous stage and according to the participants’ signals detected using the strain-based pulse wave sensor and the PPG sensor. BP values were measured before and during exercise and after a 5-min rest.

**Figure 13:**
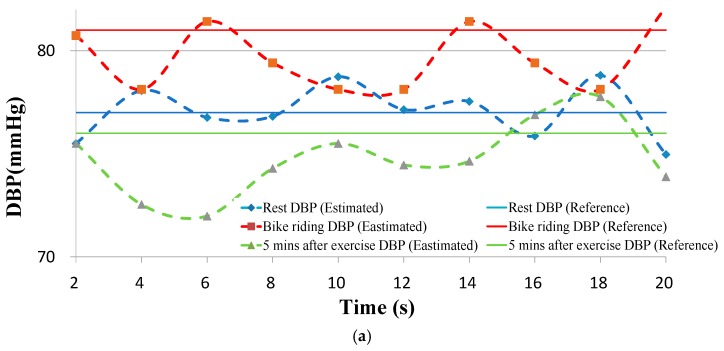

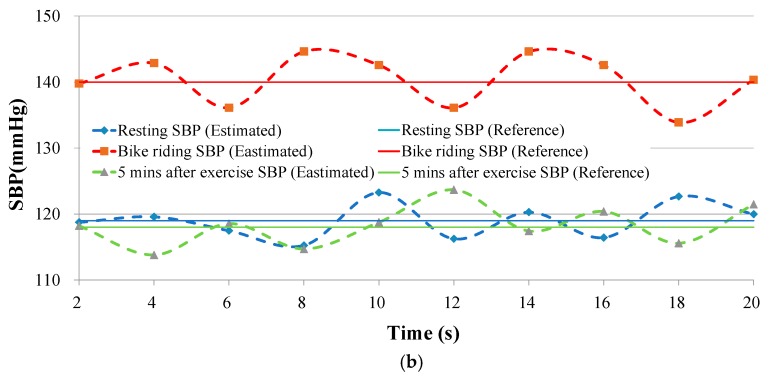
Estimated and reference BP versus time. (**a**) DBP and (**b**) SBP.

The BP data from the clinically validated sphygmomanometer were compared with those obtained from the proposed BP estimation method. Continuously estimated and reference DBPs versus time for an individual are presented in Figure 13a. The estimated BPs varied with strain and PTT values. The SBP results are presented in Figure 13b. The participants were 39 East Asian people aged 14–49 years. In the measurements, the magnitude and location of the pressing force was monitored using the pressing force sensor to ensure the consistency of the pressing force. Each participant experienced two measurement cycles in the experiment. Because a linear regression was used for parameter calibration, the difference between the two calibrated BP values should have been sufficient to minimize the calibration error. The during-exercise BP data for calibration were acquired during participants’ engagement in gentle exercise (substantial increases in BP values were not required). These procedures were sufficient for accurate estimations. To verify the device’s capacity to measure accurately large changes in BP, we added a new group of participants, which comprised 20 adults. Their BP models were calibrated according to measurements obtained during rest and gentle exercise. However, these participants engaged in high-intensity bike riding to increase their SBPs to verify the accuracy of the estimations.

## 5. Results and Discussion

**Figure 14:**
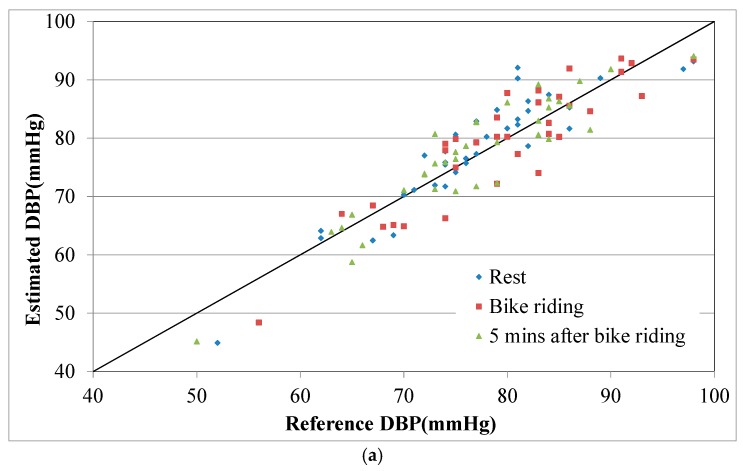

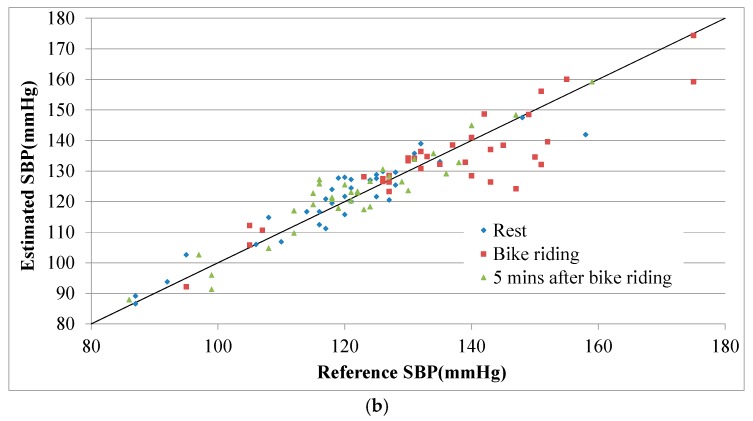
Scatter plot of participants’ reference and estimated BPs. (**a**) DBP and (**b**) SBP.

Table 1 lists the estimation errors in and averages of the estimated BPs for the 39 participants. Before the bike-riding exercise, the DBP and SBP errors for the participants’ data were both small. For the data collected during the bike-riding session, the SBP error increased, which was attributed to fact that SBP typically changes during exercise. For the data collected after participants’ 5-min rest, both the DBP and SBP errors were higher than those for the initial rest period. At this stage, some participants’ radial arteries had moved slightly out of the strain sensor’s range because of the exercise, which caused higher estimation errors. The estimated and reference BP values for all participants are plotted in Figure 14. Most of the estimated and reference BP values were well correlated, and the errors were independent from the BP values.

In the high-intensity exercise group, the respective DBP and SBP differences between values obtained during rest and exercise were 4.52 and 12.11 mmHg in the calibration. The participants then engaged in high-intensity bike riding, increasing their SBPs to an average of 152.28 mmHg. The errors and averages of the estimated BPs for the 20 participants are listed in Table 2. The mean and average errors for BP values increased slightly both during and after bike riding, as denoted in Table 1 and Table 2. A percentage error index was defined as Equation (10) to determine whether the percentage error, *E_p_*, of the estimation model increased with BP.

(10) $$E_{p}=\frac{\mathrm{Mean}\;\mathrm{absolute}\;\mathrm{error}\;\mathrm{of}\;\mathrm{BP}}{\mathrm{Average}\;\mathrm{estimated}\;\mathrm{BP}}$$

The *E_p_* values for SBPs associated with gentle exercise test (Table 1) and high-intensity exercise test (Table 2) were 5.28% and 6.92%, respectively. Similar results were observed for DBP. These findings confirmed that the proposed estimation model facilitated accurate estimation of BP. The high-intensity exercise may have induced position shifts during measurement and may have caused participants to sweat, influencing contact between the skin and strain gauge to enlarge the error.

## 6. Conclusions

The proposed strain-based pulse wave sensor and a PPG sensor could detect pulse wave signals (from the radial artery of the wrist) and PPG signals (from the finger pulp). An equation, expressing a relation of skin strain and PTT with BP was derived based on a physical model for wrist tissues and vessels, was employed to estimate BP values. The strain-based sensor was combined with a pressing force sensor to ensure consistent BP measurement conditions, enabling continuous BP monitoring without condition-related limitations on measurement. Thus, the proposed method may be used to address health care needs. In the experimental study, BP waveforms were measured before, during, and after exercise and then compared. The percentage errors defined by Equation (10) for DBP and SBP and based on the data used in Table 1 and Table 2 were, respectively, 4.74% and 4.49% before exercise, 6.38% and 6.10% during exercise, and 5.98% and 4.81% after the participants had rested. For measurements obtained after exercise, the DBP and SBP error values were lower than those associated with measurements obtained during exercise. Moreover, the proposed method based on calibration using BP data acquired from the rest and gentle exercise conditions can estimate BPs exceeding the calibration range. These results verified the applicability of the proposed BP estimation method. In the future, further details about the participants (e.g., height, weight, body mass index, and age) could be collected to assess the influence of these factors on BP estimation. Regarding its applications, the proposed system may be used to monitor sudden abnormal BP and for continuous monitoring of patients receiving long-term care (which may be helpful to the medical professionals or family members responsible for such patients). Moreover, the proposed system could be combined with smartphone applications to facilitate real-time physiological data collection and display. Users would be able to measure their BP statuses by attaching a PPG sensor (embedded in a smartphone) to their finger pulp and wearing a wrist belt sensor.

## Figures and Tables

**Table 1:** Averages and errors of the estimated BPs.

State	DBP Error	SBP Error
Rest	3.71 ± 3.06 (77.37)	5.44 ± 5.10 (122.42)
Bike riding	4.84 ± 3.65 (81.37)	7.04 ± 6.40 (133.43)
5 mins after bike riding	4.22 ± 3.98 (77.62)	5.55 ± 5.16 (122.80)

(Mean absolute error ± standard deviation of the absolute error; unit: mmHg), (average value of estimation; unit: mmHg).

**Table 2:** Averages and errors of the estimated BPs for participants who engaged in high-intensity bike riding.

State	Diastolic BP (DBP) Error	Systolic BP (SBP) Error
Rest	3.56 ± 3.01 (76.27)	5.40 ± 4.95 (118.82)
High-intensity bike riding	5.68 ± 4.02 (83.58)	10.54 ± 7.96 (152.28)
10 mins after bike riding	4.96 ± 4.21 (75.52)	6.08 ± 5.66 (119.21)

(Mean absolute error ± standard deviation of the absolute error; unit: mmHg), (average value of estimation; unit: mmHg).
